# Is acceptance and commitment therapy helpful in reducing anxiety symptomatology in people aged 65 or over? A systematic review

**DOI:** 10.3389/fpsyt.2022.976363

**Published:** 2022-10-14

**Authors:** Iraida Delhom, Joaquín Mateu-Mollá, Laura Lacomba-Trejo

**Affiliations:** ^1^Faculty of Health Sciences, Valencian International University, Valencia, Spain; ^2^Departament of Personality, Assessment and Psychological Treatment, Faculty of Psychology and Speech Therapy, Universitat de València, Valencia, Spain

**Keywords:** anxiety, acceptance and commitment therapy, systematic review, older adult, aging

## Abstract

Anxiety-related mental health problems constitute a health challenge, especially in the elderly population. At present, there are few psychological treatments to reduce anxiety adapted to this group. The aim of this study was to conduct a systematic review of the literature to determine the therapeutic effects of Acceptance and Commitment Therapy (ACT) on anxiety in older adults, using the Preferred Reporting Items for Systematic Reviews and Meta-Analyses (PRISMA) protocol. Two blinded reviewers participated in the search, selection and methodological quality assessment processes; reaching satisfactory levels of agreement between reviewers (κ > 0.70). The search was performed in PubMed, Proquest Central, Scopus and Web of Science; making use of standardized terms for the construction of the algorithm. In the general search 348 studies were found. After applying the eligibility criteria and excluding duplicates, seven articles were extracted for qualitative analysis. The total number of subjects was 633, with an average age of 68.89 years (68.94% women). The analysis of methodological rigor showed moderate indices on average. The publications focused primarily on samples with a diagnosis of generalized anxiety disorder, proposing a variety of assessment tests for related dependent variables, especially depressive symptoms and psychological flexibility. Critical analysis of the findings provides evidence for the efficacy of ACT in reducing anxious and depressive symptoms in older adults. This study proposes the use of this procedure as a non-pharmacological alternative for a group usually underrepresented in the scientific literature on this topic.

## Introduction

Anxiety-related mental health problems in older adults are among the most persistent, prevalent and impactful (1–3). However, even today, their prevalence is not yet established. Values for anxiety-related mental health problems range from 1.20 to 15%, while rates of clinically significant anxiety symptoms vary from 15 to 52% (4). It has been found that anxiety disorders tend to be persistent in older adults, with an average duration of 20 years or more (5). Furthermore, there appears to be a bidirectional association between anxiety and disability (2, 6): anxiety increases disability and worsens both quality of life and life satisfaction (4) and is associated with an increased risk of mortality in older adults, both from suicide and physical illness (7). Besides, anxiety is considered a risk factor for cognitive impairment in cognitively normal older adults (8). In fact, there is evidence that suggests that anxiety in older adults may lead to mild cognitive amnestic impairment (8). Despite the above, few studies have focused on developing psychological treatments adapted to older people (9).

The tendency toward fear and avoidance of internal experiences is a relevant feature of people with anxiety (10). Experiential avoidance is defined by Acceptance and Commitment Therapy (ACT) as one of the psychological inflexibility processes associated with psychopathology (11). According to Hayes, et al. (11), the six processes associated with psychological inflexibility and consequently psychopathology are experiential avoidance, cognitive defusion, dominance of the conceptualized past or future, disengagement from personal values, impulsivity, and persistent avoidance. Research suggests that these core psychopathology processes persist into adulthood, with a significant association between experiential avoidance of distressing internal experiences and increased anxiety in later life (12). People who display psychological inflexibility are considered to exert energy and resources toward experiential avoidance, as well as neglecting and disengaging from core values in their lives (12). Kashdan, et al. (13) used a 21-day experience sampling methodology to examine the relationships between experiential avoidance, suppressing emotions, and cognitive reappraisal with daily reports of social anxiety. Including cognitive reappraisal allowed the comparison of a core process of traditional cognitive-behavioral therapies with the core process of acceptance and attention-based therapies, experiential avoidance or acceptance. The results showed that people who worked with ACT intervention techniques reported a significant reduction in anxiety levels. ACT-based treatment aims to focus attention on feeling better and living better. These authors consider ACT-based exercises to be more effective than cognitive behavioral therapy as they are not portrayed as a way to reduce anxiety, but as a strategy to develop a willingness to deal with anxiety while progressing toward a set of desired goals (13). Similar studies have shown how thought suppression as a mechanism to combat unwanted thoughts has been associated with a less subjective meaning of one's life in older adults (14). Petkus & Wetherell, et al. (12) confirmed how thought suppression was associated with more somatic, depressive and anxiety symptoms after physical, functional and cognitive disease control in a sample of older adults with functional disability and chronic illness (12). In the same line, more recent research exploring ACT in older adults suggests that ACT contributes more significantly to alleviating anxious symptoms than traditional cognitive-behavioral techniques (2, 15, 16).

Numerous reasons support the appropriateness of ACT in older adults (9, 10, 17). On the one hand, anxiety disorders often present a chronic condition resulting in their onset prior to old age and their persistence over time (5). In addition, anxiety disorders often show greater resistance to treatment in older adults (9). On the other hand, comorbidity of anxiety and depression are common in older adults, making them more difficult to differentiate (18). The transdiagnostic nature of ACT makes assessment and intervention for anxiety and depression more efficient, as it is not necessary to distinguish between both pathologies, as it focuses exclusively on determining how core processes of psychological inflexibility contribute to psychopathology in order to intervene on them, regardless of the current mental health problem (12). In addition, ACT can be beneficial in cases where psychological distress is associated with loss-related factors that are unavoidable and immutable. In this way, the acceptance approach and refocusing behaviors on attainable goals aligned with one's values can be particularly beneficial (17).

In summary, the particularities and challenges presented by anxiety in older adults require special attention to address this issue in an idiosyncratic manner. ACT has shown to be potentially successful in this regard; however, more evidence is needed to support these findings so far, especially in older adults. Therefore, this study aimed to conduct a systematic review in order to compile the available evidence on the efficacy of ACT in older adults with anxiety problems.

## Methods

This systematic review was developed according to the Preferred Reporting Items for Systematic Reviews and Meta-Analyses (PRISMA) (19).

### Bibliographic search

The Proquest, Pubmed, Web of Science and Scopus databases were consulted by two authors (LL-T and JM-M), exploring articles published before 29th May 2022. Using the PICO approach (20), the following research question was posed: do older people who undergo ACT improve their anxiety levels?.

The searching protocol was applied to all the selected databases and was constructed as follows: “acceptance and commitment therapy” AND (anxiety OR “anxiety disorders”) AND (aged OR aging). In order to optimize the process, standardized terms were retrieved from the Medical Subject Headings—MeSH (English) and from Descriptores en Ciencias de la Salud—DeCS (Spanish).

All selected articles were managed through the Covidence *software*. First, duplicate articles were eliminated, after which the two authors (LL-T and JM-M) reviewed the manuscripts with emphasis on the title and abstract, determining compliance with the eligibility criteria separately. In this process, articles were screened independently and in a blind manner with regard to the other author's decision. If there were conflicts, a second in-depth reading was performed individually. Finally, disagreements were resolved through active discussion. A third reviewer (IDL) provided arbitration in cases where consensus could not be reached.

**Figure 1:**
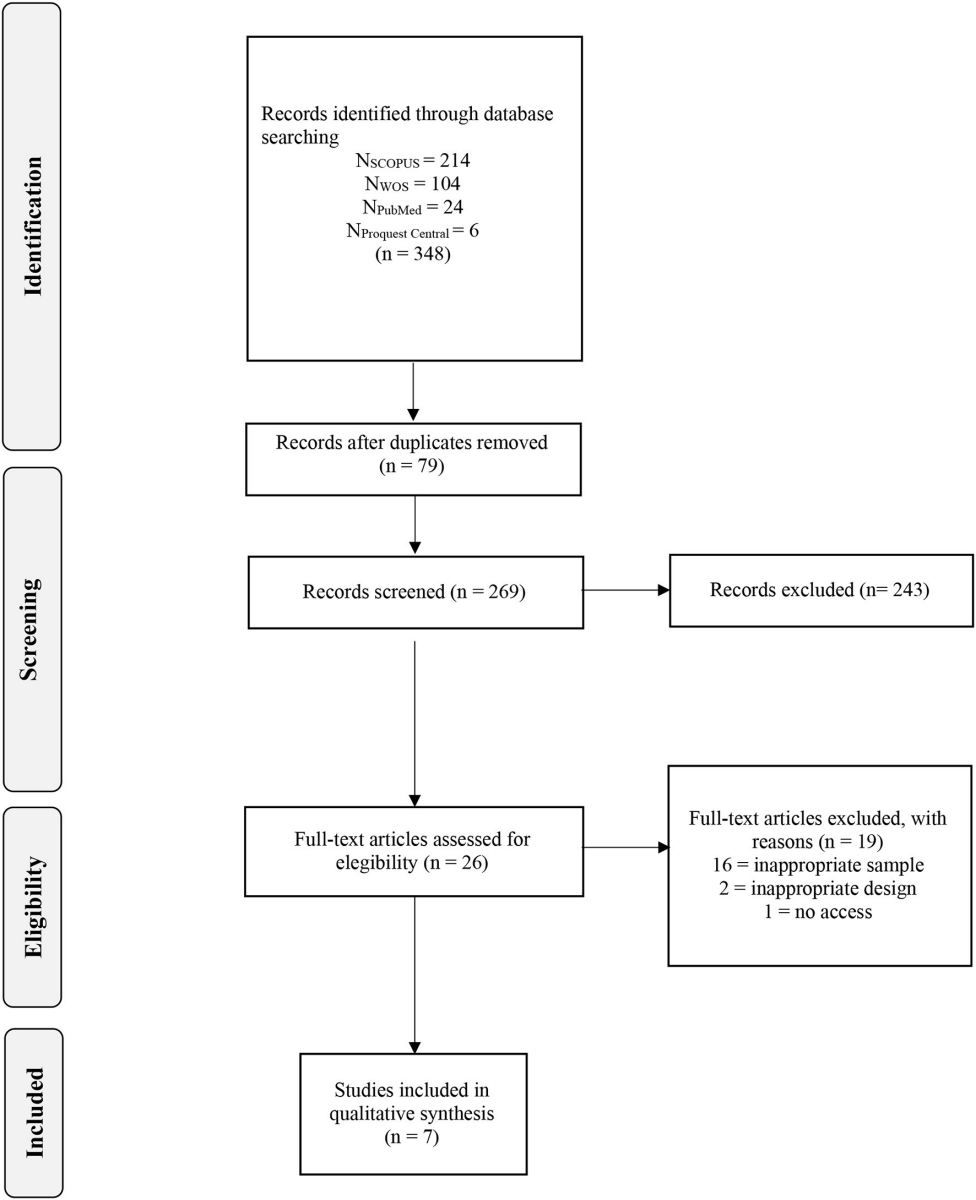
Flowchart of selection process.

The Cohen's Kappa index (κ) (21) was used to evaluate the agreement between judges; values between−1 and.40 are considered unsatisfactory, those between 0.41 and 0.75 are considered acceptable, and those that score 0.76 or higher are considered satisfactory (22). Figure 1 displays the flowchart that depicts the selection process.

### Inclusion and exclusion criteria

The inclusion criteria were: (a) the study assessed the benefits attributable ACT on the anxious symptomatology, (b) the age of the sample was 65 years or older, (c) the manuscript underwent a peer review process, (d) the article was published in a journal with significant impact factor, (e) the study was published in English or Spanish, and (f) the paper was published within the last 10 years.

The following exclusion criteria were agreed upon: (a) samples of patients diagnosed with schizophrenia and bipolar disorder, (b) samples of patients diagnosed with severe medical conditions, (c) publications derived from conferences, (d) studies based on a narrative review, and (e) studies that did not explore anxiety using a scientifically validated questionnaire.

### Data collection

One of the authors (LLT) developed an *ad hoc* table to synthesize all relevant information from the selected articles. This information included: (a) first author, (b) year of publication, (c) source country, (d) objectives, (e) sample, (f) variables and instruments, (g) design, (h) intervention, (i) results, (j) main conclusions, (k) methodological rigor indices, and (l) dropouts (Table 1).

**Table 1:** Characteristics of studies included in the systematic review (*N* = 7) and article quality assessment.

**First author**	**Year**	**Country**	**Objectives**	**Sample**	**Variables and instruments**	**Design**	**Intervention**	**Results**	**Conclusions**	**Dropouts**
**Davison**	2016	Australia	- Assess the efficacy of ACT to improve anxiety and depression.	41 older adults 63–97 years (85.33 ± 9.20) (85.24% women)	- Sociodemographic (sex, gender, time of residence). - Clinical (medical files baseline and medication). - Psychological: depression (Geriatric Depression Scale (GDS-15) (23), and The Cornell Scale for Depression in Dementia (24), anxiety (Geriatric Anxiety Inventory (GAI) (25). - Treatment satisfaction in participants (Client Satisfaction Questionnaire; CSQ-8) (26) and staff (*ad hoc*).	Descriptive non-experimental, longitudinal	−12 individual sessions of 60 min (two per week). - Implemented by PhD students with training in psychotherapy and older people.	- After the intervention, depression was reduced, but not anxiety. - These improvements were maintained after 3 months. - It was observed that both participants and healthcare staff found the treatment useful and expressed satisfaction.	- ACT appears to be helpful for older people in reducing depression. - It has not been proven to be successful in reducing anxiety. - It appears to be a well-received treatment by participants and health workers.	50 %
**Fowler**	2021	United States	- To assess the feasibility, acceptability, and preliminary effects of an ACT intervention on ADRD caregiver anxiety, depressive symptoms, burden, suffering, and psychological flexibility.	15 participants aged >21 (68.85 ± 11,70) (80.00 women)	- Sociodemographic: age, sex, ethnicity, education, income, place of recruitment, relationship of the caregiver and the patient with ADRD, severity of ADRD. - Psychological: Anxiety (Generalized Anxiety Disorder Scale; GAD-7) (27), Depression (Patient Health Questionnaire-9; PHQ-9) (28), Caregiver burden (Zarit Burden Interview; ZBI) (29), Cognitive Flexibility (Acceptance and Action Questionnaire-II; AAQ-II) (30) Coping (Brief COPE) (31).	Descriptive non-experimental, longitudinal	−6 Telephone-based sessions of 60 min (one per week) - Implemented by a bachelor's-level interventionist with a 4-year degree in Psychology but not a licensed therapist or psychologist that received supervision throughout the study from a Master's level clinician with ACT training	- After the intervention anxiety was reduced, but not depression - The improvements were maintained at 6 months. - At 6 months, statistically significant decreases in caregiver psychological suffering and caregiver burden were observed	- ACT seems useful for older people in reducing anxiety. - ACT could improve caregiver psychological suffering and caregiver burden. - Benefits can be maintained over time.	6.25 %
**Gould**	2021	United Kingdom	- To examine the acceptability, feasibility, and preliminary estimates of the effectiveness of ACT for older people with generalized anxiety disorder	37 older adults aged >65 (74.80 ± 6,30) (81.00% women)	- Sociodemographic: age, sex, ethnicity, marital status, mean years of education, highest educational qualification, employment status. -Psychological: Anxiety (Geriatric Anxiety Inventory (GAI) (25) and Penn State Worry Questionnaire (PSWQ) (31), Depression (Geriatric Depression Scale (GDS-15) (23), Cognitive Flexibility (Acceptance and Action Questionnaire-II (AAQ-II) (30), -Cognitive: Standardized Mini-Mental State Examination (SMMSE) (51). -Satisfaction: Satisfaction with Therapy and Therapist Scale-Revised (48).	Descriptive non-experimental, longitudinal	- 16 individual 60-min ACT sessions. The first 14 sessions were weekly, while the following sessions were fortnightly to facilitate the completion of the intervention. -Therapists were qualified clinical psychologists, cognitive-behavioral therapists or counseling psychologists, with a minimum of 1 year of experience in conducting psychotherapy interventions.	- Improvements in anxiety, depression and the acceptance and action questionnaire. - The improvements were maintained at 20 weeks. - Adequate satisfaction with therapy.	- There was excellent evidence of feasibility and good evidence of acceptability of ACT for older people with generalized anxiety disorder. However, satisfaction with therapy scores suggested that further refinement of the intervention may be necessary.	24.32 %
**Jacobs**	2018	United States	- To develop, implement, and evaluate a 12-session ACT for older veterans group protocol.	17 older adults aged 55–84 (68.00 ± 6.59) (100% men)	- Sociodemographic: sex, race, ethnicity, marital status. - Clinical: diagnosis of anxiety disorder. - Psychological: Anxiety (Generalized Anxiety Disorder Scale; GAD-7) (27), Cognitive Flexibility (Acceptance and Action Questionnaire-II	Descriptive non-experimental, longitudinal	−12-session ACT of 60 min, that included brief homework assignments (one per week) - Therapist were working in an outpatient geropsychology	- After the intervention, depression was reduced, but not anxiety	- Results provide support for the use of ACT in the treatment of depressive symptomatology among older adults - Results failed to show significant	23.52 %
					(AAQ-II) (30), Depression (Geriatric Depression Scale (GDS-15) (23).		clinic at a rural Veterans Affairs Medical Center		changes in anxiety.	
**Lappalainen**	2021	Finland	- To develop and investigate whether a novel ACT-based online intervention effectively enhances the psychological well-being of family caregivers aged 60 and over.	149 older adults aged >60 (72.90 ± 6, 10) (80.50% women)	- Sociodemographic: sex, marital status, education, caregiver diagnosis, care recipient, age of the care recipient, number of years providing care, receiving of care allowance. - Psychological: Depression (Beck Depression Inventory—II; BDI-II) (32), Anxiety (Generalized Anxiety Disorder Scale; GAD-7) (27), Quality of Life (WHOQOL-BREF Quality of Life Survey) (33), sense of coherence (SOC) (34), Psychological Flexibility (Acceptance and Action Questionnaire-II (AAQ-II) (30), Experiential Avoidance in Caregiving Questionnaire (EACQ) (52), White Bear Suppression Inventory (WBSI) (50).	Descriptive non-experimental, longitudinal	−12 web-based ACT sessions of 90 min, one per week, divided into six progressive intervention modules on ACT processes enhanced with a compassion module (group 1) - Standardized institutional rehabilitation provided by rehabilitation centers during 10 months (group 2).	- Web-based ACT sessions were better than standardized institutional rehabilitation for reducing depressive symptoms and enhancing the psychological quality of life. - No statistical differences were found between groups for anxiety, physical quality of life, social quality of life, environmental quality of life, and sense of coherence.	- Online Care ACT intervention produced significant effects on depressive symptoms over the duration of the intervention (12 weeks), and the CareACT intervention was superior to the comparison interventions. - Treatment effects were not fully maintained at 10 months.	28.86 %
**Chojak**	2021	Poland	- To assess the effectiveness of psychological skills training based on ACT in lowering the psychopathological symptoms and increasing the quality of life in older adults over 60 years of age.	60 older adults aged 60–91 (73.48 ± 8.87) (80.00% women)	- Sociodemographic: age, sex, education level. - Psychological: Depression, Anxiety and Stress Scale (DASS-21) (35), WHO Quality of Life—AGE Scale (WHO-QOL-AGE) (36). - Cognitive: Mini Mental State Examination (MMSE) (37).	Descriptive non-experimental, longitudinal	−12 ACT sessions of 60 min (three-four per week) (intervention group; *N* = 30). - Passive (Control Group; *N* = 30).	- After the training, the level of anxiety symptoms in the experimental group was significantly reduced. - Simple effect analysis for the measurement showed no significant difference in the level of depressive symptoms in the control group, while in the experimental group, the difference was significant. - Simple effects analysis for the measurement showed no significant difference for the control group, while there was a significant difference between measurements in the experimental group.	- The results of statistical analyses confirmed the hypothesis about the effectiveness of ACT based training of psychological competencies in lowering the symptoms of psychopathology and improving the quality of life. - ACT-based programes can be implemented for older adults at risk of social exclusion or suffering from higher levels of psychopathological symptoms.	0 %
**Wiltox**	2021	Netherland	- To examine the effectiveness of a combined brief ACT intervention for older adults with anxiety symptoms compared to a face-to-face CBT intervention.	314 older adults aged 55–75 (62.75 ± 5.69 in ACT group and 63.33 ± 5.71 in CBT group) (66.70% women in ACT group and 56.10% women in CBT Group)	- Sociodemographic: age, sex, nationality, education, relational status, work status, living situation, - Clinical: somatic comorbidity, use of psychomedication and anxiety disorder. - Psychological: Anxiety (Generalized Anxiety Disorder Scale; GAD-7) (27), Depression (Patient Health Questionnaire-9; PHQ-) (28), Mental Health Continuum Short Form (MHC-SF) (38), Disability (Sheehan Disability Scale; SDS) (39), Personality (MINI-Plus) (49). - Satisfaction: Client Satisfaction Questionnaire (26)	Randomized Controlled Trial	- Living to the Fullest Programme (ACT group): 9 lessons (15–30 min) to be completed in 9–12 weeks. The web-based module can be accessed *via* computers and mobile devices. - Standard treatment: Face-to-face CBT (CBT group): 4 face-to-face sessions over a period of 9–12 weeks. Participants received homework exercises requiring 15–30 min per day (a similar time investment to the ACT intervention).	- Regardless of the condition, GAD-7 scores decreased significantly from T0 to T1, increased significantly between T1 and T2, and did not change significantly from T1 to T3. - MHC-SF scores decreased in the CBT group, whereas they increased in the ACT group. - Regardless of the condition, depression severity decreased over time. - Functional impairment in work, social life and family life decreased significantly from baseline to post-treatment in all groups. These improvements were maintained at the one-month follow-up.	- No differences between blended ACT and face-to-face CBT in their effects on anxiety symptom severity. - In both groups, anxiety symptoms improved significantly from baseline to posttreatment. - Blended ACT is a valuable treatment alternative to CBT for anxiety in later life.	39.17 %
								- Treatment satisfaction was significantly higher in the ACT group than in the CBT group, and the effect size of the difference was large.		
**Article quality assessment**										
**First author**	**Study design**	**Blinding**	**Representativeness selection bias**	**Representativeness withdrawals and dropouts**	**Confounders**	**Data collection methods**	**Data analysis**	**Reporting**	**Total**	**Overall quality assessment**
Davison	2	3	3	2	1	1	1	1	1.38	Weak
Fowler	2	3	3	1	1	1	2	2	1.88	Weak
Gould	2	3	3	2	1	1	1	2	1.88	Weak
Jacobs	2	3	3	2	1	1	1	1	1.88	Weak
Lappalainen	2	3	3	2	1	1	2	2	2.00	Weak
Chojak	2	3	3	1	1	1	2	2	1.88	Weak
Wiltox	1	1	1	2	1	1	2	2	1.38	Strong

### Quality assessment

Two authors (LL-T and JM-M) assessed the methodological rigor of the selected studies in an independent and blinded manner using an adapted version of the Quality Assessment Tool for Quantitative Studies developed by the Effective Public Health Practice Project (40). This tool consists of 19 items that assess 8 criteria: (a) study design, (b) blinding, (c) selection bias, (d) withdrawals and dropouts, (e) confounders, (f) data collection methods, (g) data analysis, and (h) reporting. Studies can have between 4 and 8 component ratings based on these criteria (41). The average quality score was 1.84, with quality scores ranging from 1 to 3, with 1 being the highest score (least likely to be biased and highest quality) and 3 being the weakest score (most likely to be biased or lowest quality). A study with 6 ratings could be rated as “strong” if there are no weak ratings and at least 3 strong ratings, “moderate” if there is one rating and <3 strong ratings, or “weak” if there are two or more weak ratings.

## Results

### Study selection and screening

The study selection process is shown in Figure 1. The literature screening resulted in a total of 348 records. After removing duplicates, the total number of records was 77. The initial selection excluded 189 studies based on title and abstract, and the full content of the remaining 42 papers was read as part of a second selection process. The reliability of prior agreement between the two independent reviewers (LL-T and JM-M) in the full-text selection was excellent (κ = 0.84). In the second screening, 30 papers were excluded resulting in 7 dependent studies being eligible for inclusion. The degree of agreement between the reviewers was also excellent (κ = 0.82).

### Characteristics of the study

The characteristics of the studies are summarized in Table 1. The seven studies investigated included a total of 633 older adults. The samples ranged from 15 participants to 134, with an average sample size of 90.43. Of the participants, 68.94% were female. The age of participants ranged from 57.14 to 94.22 years, with an average of 68.89 years. All studies were longitudinal. However, only two of them included a control group; one had the same control group as the experimental group (waiting list), and in the other, the control group received CBT. Three of the studies had at least one follow-up. The mean assessment time in these studies was 6.86 weeks for the first measurement, ranging from 6 to 24 months between assessments. The mean number of weeks at follow-up was 24.60.

Regarding the sample's representativeness for treatment dropout, all but one study reported this information. This percentage ranged from 6.25 to 39.17. The mean dropout rate from the studies was estimated at 24.66%. Futhermore, although most papers do not mention the reason, they are generally high. In those that do mention the reason for dropout, it is said to be due to difficulties in following treatment or death. There was considerable heterogeneity in the independent and dependent variables assessed (χ^2^ = 8.55, *p* = 0.010, *V* = 0.45) as well as in the number of participants (χ^2^ = 10.00, *p* = 0.007, *V* = 0.60). However, the study designs were acceptably heterogeneous (χ^2^ = 0,09, *p* = ,520, *V* = 0,01). Most studies assessed anxiety, depression, stress or burden, and ACT elements such as cognitive flexibility. Other variables of interest were personality, psychopathology, life satisfaction, cognitive impairment and quality of life. Four studies report that the therapy was conducted by qualified psychologists, while the rest do not provide any information. Most interventions lasted for about 12 weeks, but in one case (42), treatment lasted for 6 sessions, and in another (15), it lasted for 16 sessions. There was also heterogeneity in the presentation of the therapies. Overall, 4 studies were individual and face-to-face, while one study (42) was conducted over the telephone and two studies were conducted over online modules. In this regard, the two reviewers (LL-T and JM-M) assessed the presence or absence of a control group and the presentation format, with inter-rater reliability reaching an almost perfect level of agreement (κ = 90). Every study controlled at least one confounding variable (medication intake, type of medication, caregiver relationship, gender, background, session attendance, waiting time between treatment and assessment, marital status, education, employment status, mental health status, and socio-demographic and clinical variables), and they all mention inclusion and exclusion criteria. In general, weak statistical tests were used. However, two studies (15, 43) did use ANCOVA to evaluate the programmes.

## Discussion

This study aimed to conduct a systematic review to compile the available evidence on the efficacy of ACT in older adults with anxiety problems. A total of 7 papers were included. Overall, 57.14% of the studies focused on the population with generalized anxiety disorder, 28.57% on caregivers of dependent persons and 14.29% on anxious and depressive symptoms associated with long-term institutionalization.

These studies showed how ACT was effective in reducing depressive (15, 43–47), and anxiety symptoms (15, 42, 44, 47). Regarding anxiety, the results are less consistent, with studies concluding that ACT could reduce anxiety symptoms (15, 42, 44, 47) and other studies finding that it does not (43, 45, 46). One of the studies (47) compared the benefits of ACT with those of traditional CBT, concluding that both procedures effectively reduce symptoms of anxiety and depression. However, ACT showed a superior therapeutic impact on mental health and treatment satisfaction. For both ACT and CBT, the effects were maintained at follow-up. Another paper (46) compared ACT treatment conducted online with the traditional approach provided by rehabilitation centers, finding that the online modality of ACT was more effective in reducing depressive symptoms and strengthening the psychological components of quality of life.

The findings of the reviewed studies have some limitations. Some of the studies have been conducted with very small samples (42, 45). Furthermore, there is a lack of control and experimental groups (15, 42, 43, 45, 46), and only one randomized controlled trial was found (47). On the other hand, the available studies assess anxiety in various ways, in many cases alongside other emotional problems. In 25% of the studies, the GAI was used (25), 50% used the GAD-7 (34), 12.50% used the DASS-21 (35) and the remaining 12.50% used *ad hoc* semi-structured interviews. In addition to the above, most of the studies included have high drop-out rates of over 20%. Not all studies provide reasons for the dropouts. Finally, despite having performed a heterogeneity analysis, results should be considered cautiously. This type of analysis should be conducted in more endline studies.

However, preliminary results indicate that ACT can be beneficial for treating anxiety problems in older adults, particularly suitable for this population's characteristics (2, 15, 17). Therefore, these tools should be studied in greater depth to maximize their benefits. In this regard, further research of higher quality is required, including additional randomized controlled trials specifically targeting older people.

The results of this research may be affected by some biases, including the following: biases in the sample size, as well as in the research design (non-randomization of participants, general lack of control and experimental group, no double-blind allocation between control and experimental group; use of weak statistics and general lack of follow-up), assessment biases (selection of non-specific assessment instruments for older adults), and sampling biases.

On the other hand, the limited selection of articles directly related to the systematicity of the review requires further research. In addition to the above, most articles address the benefits of ACT in older people with a diagnosis of generalized anxiety disorder, but other clinical conditions of the same psychopathological category are underrepresented. Furthermore, there has been a significant heterogeneity of assessment methods and therapeutic procedures. Although the number of sessions tends to be similar among the studies; their contents, timing and administration methods are highly variable, making it impossible to carry out a meta-analysis at the present time. Similarly, the quality of the studies analyzed has been found to be moderate or weak.

To the best of our knowledge, this is the first systematic review conducted on the possible benefits of ACT on anxiety in older adults. Based on the results obtained, it can be concluded that there is a need for further higher-quality research focused on this area that would allow to truly evaluate the benefits of ACT in older adults.

## Author contributions

ID: project management, literature search, writing the original manuscript, and revising the manuscript. JM-M: review proposal, conducting the search process, methodology, review and synthesis of the review articles, and writing the original manuscript and revision. LL-T: review proposal, conducting the search process, review and synthesis of the review articles, and statistical analysis and revision of the manuscript.

## Funding

Research Project Emotional intelligence as a resource for successful adaptation in everyday life (PII2021_06), funded by Valencian International University. LL-T is a beneficiary of the Ayuda de Atracció a Talent de la Universitat de València (0113/2018).

## Conflict of interest

The authors declare that the research was conducted in the absence of any commercial or financial relationships that could be construed as a potential conflict of interest.

## Publisher's note

All claims expressed in this article are solely those of the authors and do not necessarily represent those of their affiliated organizations, or those of the publisher, the editors and the reviewers. Any product that may be evaluated in this article, or claim that may be made by its manufacturer, is not guaranteed or endorsed by the publisher.
